# A case report of COVID-19 in a patient with non-Hodgkin’s lymphoma

**DOI:** 10.1186/s12879-021-06472-2

**Published:** 2021-08-12

**Authors:** Owrang Eilami, Max Igor Banks Ferreira Lopes, Ronaldo Cesar Borges Gryschek, Kaveh Taghipour

**Affiliations:** 1 Department of Infectious Diseases and Family Medicine, Shiraz University of Medical Sciences, Shiraz, Iran; 2 Department of Infectious and Parasitic Diseases, Central Institute, Hospital das Clínicas, School of Medicine, University of São Paulo, São Paulo, Brazil; 3 Associate Professor of Infectious Diseases, Hospital das Clínicas,, School of Medicine, University of São Paulo, São Paulo, Brazil; 4 Department of Family Medicine, Shiraz University of Medical Sciences, Shiraz, Iran

**Keywords:** COVID, Lymphoma, Immunosuppressed

## Abstract

**Background:**

The current literature is scarce as to the outcomes of COVID-19 infection in non-Hodgkin's lymphoma patients and whether immunosuppressive or chemotherapeutic agents can cause worsening of the patients’ condition during COVID-19 infection.

**Case presentation:**

Our case is a 59-year-old gentleman who presented to the Emergency Department of the Cancer Institute of Hospital das Clínicas da Universidade de São Paulo, São Paulo, Brazil on 10th May 2020 with a worsening dyspnea and chest pain which had started 3 days prior to presentation to the Emergency Department. He had a past history of non-Hodgkin's lymphoma for which he was receiving chemotherapy. Subsequent PCR testing demonstrated that our patient was SARS-CoV-2 positive.

**Conclusion:**

In this report, we show a patient with non-Hodgkin lymphoma in the middle of chemotherapy, presented a mild clinical course of COVID-19 infection.

## Background

Since the first identification of COVID-19 in an outbreak in Wuhan China in December 2019 COVID-19 has quickly spread worldwide and was declared a worldwide pandemic on 11th March 2020. The clinical presentation of COVID-19 is variable with a clinical spectrum ranging from asymptomatic to life-threatening clinical presentation [1]. One would expect immunocompromised patients would be at high risk of complications from COVID-19 infection, however, there have been reports of variable clinical presentation and clinical course within this subgroup of patients [2]. Currently the data regarding risk and outcome of infection by SARS-CoV-2 in lymphoma patients is scarce and variable. Lymphoma patients present an interesting subgroup as they typically receive immunosuppressive and chemotherapy drugs during their treatment. We are presenting a case of symptomatic COVID-19 with comorbid non-Hodgkin's lymphoma who was receiving chemotherapy and subsequently recovered from COVID-19 infection.

## Case presentation

**Fig. 1:**
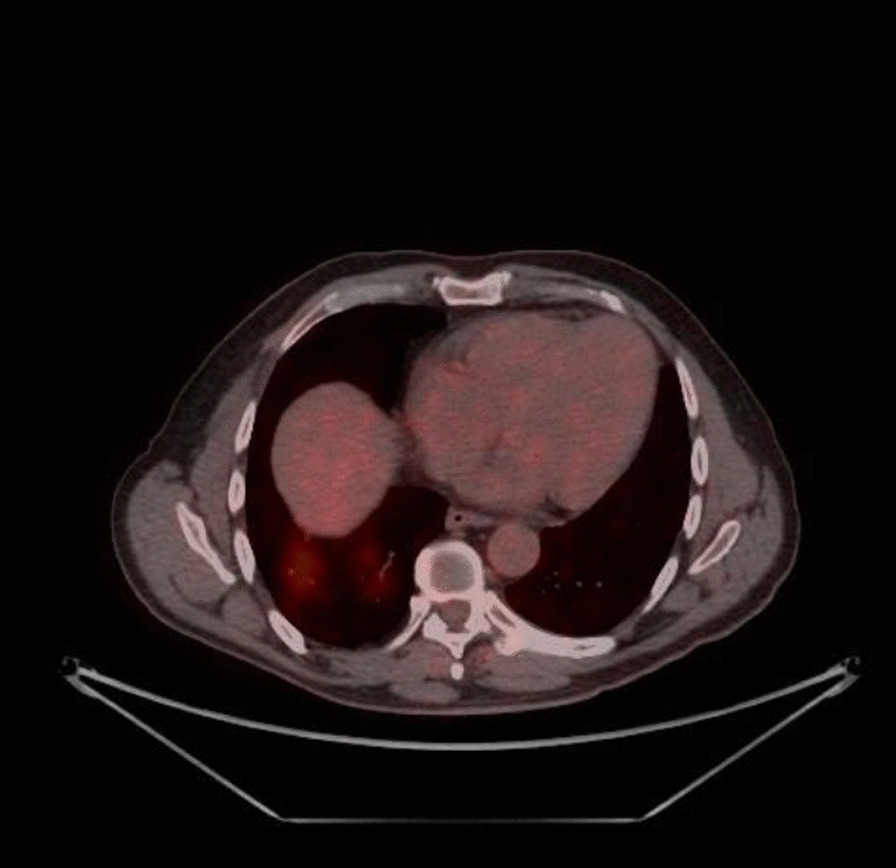
PET-CT 29/04: appearance of multiple areas of discrete opacity bilateral in pulmonary parenchyma, predominating in the base (SUVmax: 3.7-right base. The comparative analysis with a previous examination of 03/18/2020 shows: the degree of glycolytic hypermetabolism persists without significant variation associated with reduced dimensions, in heterogeneous tissue formation. The findings are compatible with uptake above mediastinal, but below or equal to uptake in the liver with nodal or extranodal sites with or without a residual mass indicating a non-progressive disease with a complete metabolic response to lymphoma treatment. The appearance of multiple areas of ground-glass opacities sparse by the bilateral pulmonary parenchyma, predominantly at the right base, suspicious for viral pneumonia

**Fig. 2:**
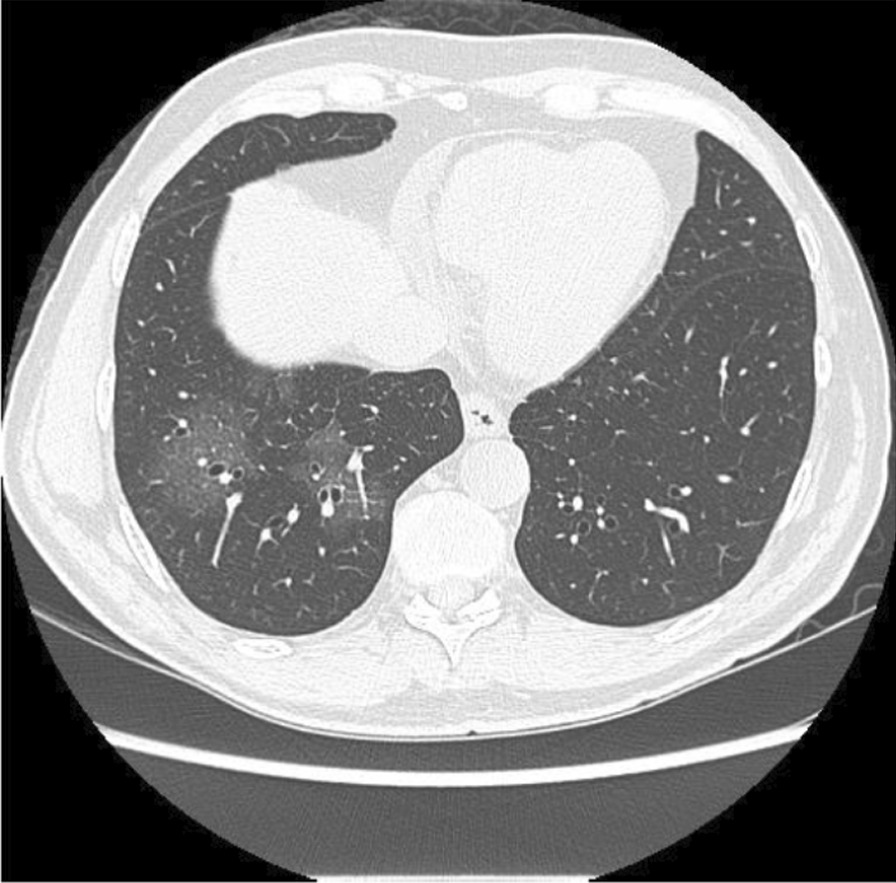
Computed tomography of the chest without the administration of the intravenous contrast medium—pulmonary ground-glass opacities and foci of consolidation, sometimes associated with septal thickening, with multifocal distribution, bilateral and predominantly peripheral. The estimated extent of involvement in pulmonary tomography is less than 50%

Our patient is a 59-year-old gentleman who presented to the Emergency Department of the Cancer Institute of Hospital das Clínicas da Universidade de São Paulo, Brazil on 10th May 2020 with worsening dyspnea and chest pain for the last 3 days. Five months prior to presentation he had been diagnosed with non-Hodgkin's lymphoma and previously, on April 29th during a routine follow-up hospital admission for his lymphoma, he had a routine PET CT (Fig. 1) and chest CT scan (Fig. 2) performed for lymphoma follow-up showing bilateral ground-glass lung lesions with less than 25% of lung involvement suggestive of COVID-19 infection. Subsequent COVID-19 PCR revealed that the patient was COVID-19 positive, however as he was asymptomatic, he was discharged.

Comorbidities presented were systemic arterial hypertension treated with losartan 50 mg bid and non-insulin-dependent diabetes treated with 60 mg of gliclazide per day and metformin 850 mg q8h.

The past medical history of our patient includes the diagnosis of lymphoma on December 1st, 2019, after an exploratory laparotomy due to perforating acute abdomen resulting in enterectomy of the perforated segment (15 cm resection) plus manual end-to-end anastomosis + Barker peritoniostomy (due to hemodynamic instability). On December 5th—a second look abdominal surgery was performed with wall closure and mesh placement. The lymphoma was classified as diffuse large cells non-Hodgkin lymphoma—CD20 negative. The patient started CHOP chemotherapy [cyclophosphamide, doxorubicin, vincristine, prednisolone] with cycles every 21 days last on April 16th and three cycles of intrathecal methotrexate (MTX) chemotherapy last on March 16th.

Eleven days following discharge from the patient's first admission, the patient’s condition worsened. Subsequently on 10th of May 2020, the patient presented with worsening cough, dyspnea and chest pain to the Emergency Department of the Cancer Institute of Hospital das Clínicas da Universidade de São Paulo, Brazil. Due to the patients deteriorating condition the patient was admitted to hospital.

**Fig. 3:**
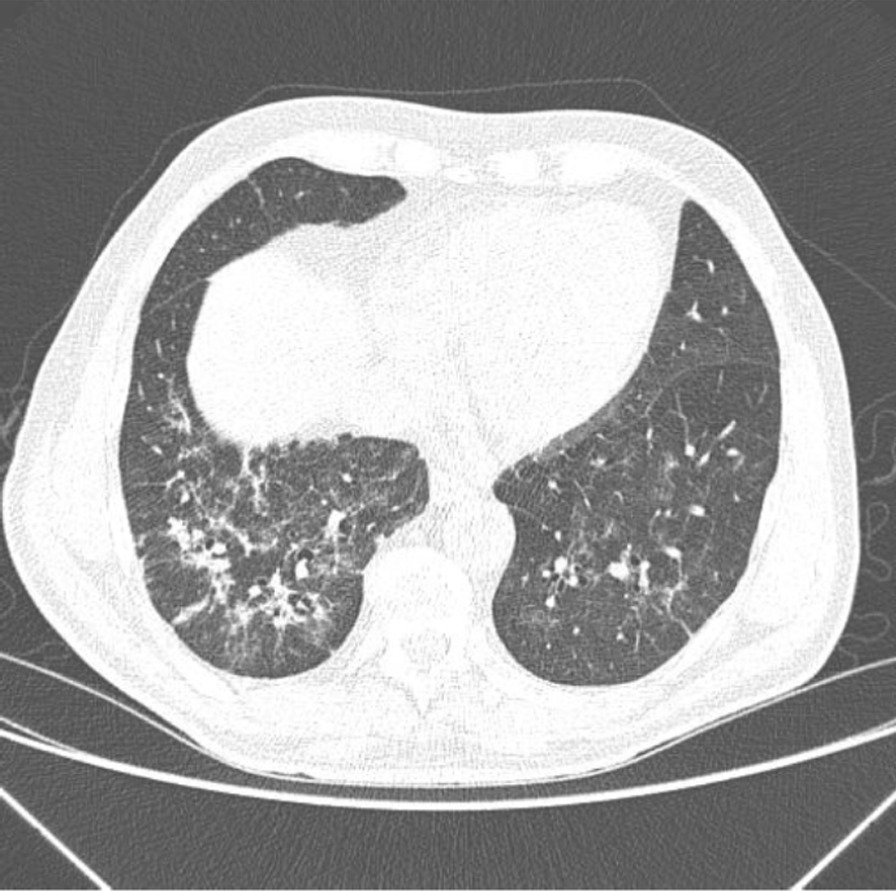
Computed Tomography of the Chest without the administration of the intravenous contrast medium—these set of findings is suggestive of the inflammatory process, and the viral etiology must be included in the etiological differential, particularly COVID-19. Regarding the PET-CT 29/04/2020 exam, there was an increase in the extension and attenuation of pulmonary opacities

During his latest hospital stay, he was prescribed ceftriaxone 2 g QD for seven days, azithromycin 500 mg QD for five days, enoxaparin 40 mg QD, oseltamivir 75 mg bid for two days (until the result of SARS-CoV-2 RT PCR positive). He received pneumocystis prophylaxis with sulfamethoxazole 400 mg + trimethoprim 80 mg and acyclovir 200 mg QD for herpes simplex prophylaxis. A second chest CT scan was performed (Fig. 3) which demonstrated an increase in the extension and attenuation of pulmonary opacities compared with the patient’s previous chest CT scan (Fig. 2). The patient’s clinical condition was stable without any worsening of clinical condition and biochemical markers (Table 1) during his hospital stay and maximum oxygen intake of 3L/min by nasal cannula. The oxygen supplementation was titrated and interrupted on May 13th. On May 19th, the patient was discharged. At that time, a second nasal SARS-CoV-2 RT PCR was performed and resulted in a negative result.

**Table 1:** Table of laboratory data values

	27/04/2020	10/05/2020	12/05/2020	15/05/2020	17/05/2020	19/05/2020
Hemoglobin	10.3	10.2	10.1	9.4	11.1	10.4
Hematocrit	32.6	32.3	30	28.4	33.5	32.1
Leucocytes	2.77	7.74	6.23	8.12	7.72	5.55
Neutrophils	57.80%	82.80%	68.70%	76.30%	71.70%	63.40%
Lymphocytes	22.40%	8.40%	17%	11.60%	17%	18.20%
Monocytes	17.30%	8.50%	12.40%	10%	10.20%	15.50%
Platelets	189	456	393	502	598	528
D-Dimer						1439
AST		15	63	24	13	
ALT		18	55	39	21	
CRP		103.5	58	92.2	31.8	11.5
BUN	28	31	21	35	35	42
Creatinine	0.83	0.78	0.73	0.8	0.82	1.01

## Discussion and conclusion

Non-Hodgkin's lymphoma presents a dilemma to healthcare professionals as there is concern that chemotherapeutic and immunosuppressive treatment which is a pillar of cancer therapy may lead to a worsening of comorbid COVID-19 infections.

The current literature is scarce as to the outcomes of COVID-19 infection in non-Hodgkin's lymphoma patients and whether immunosuppressive or chemotherapeutic agents can cause worsening of the patients’ condition during COVID-19 infection. Our case of a 59-year-old gentleman with non-Hodgkin’s lymphoma presents a glimpse and discussion of current assumptions of this subgroup of patients and comorbid COVID-19 infection because contrary to our expectations the patient had a benign clinical course without any evidence of worsening of symptoms that are contrary to our experience with other infections that are often more frequent and more severe in cancer and hematological malignancy patients.

The clinical presentation of our patient was that he had progressive dyspnea without fever. Only on the 4th day following admission the patient developed fever which is in contrast to a retrospective study by Zhang et al. conducted in Wuhan, China of 28 COVID-19-infected cancer patients which had shown that fever was present in 82.1% of patients followed by dry cough in 81% of patients and dyspnea in 50% of patients [3]. In another retrospective study by Yang et al. of 52 cancer patients with COVID-19 the common presenting symptoms were: fever (25%), dry cough (17.3%), chest distress (11.5%), and fatigue (9.6%) [4].

In a study by Chen and colleagues of 128 hospitalized patients with hematological cancer in two centers in Wuhan China it was shown that there was no significant difference in the case rate between in hematological malignancy patients versus non hematological malignancy patients [5]. Furthermore, patients with hematological malignancy had more severe disease and increase fatality when infected by COVID-19. In a study by Yigenoglu et al. of 740 COVID-19 patients with hematological malignancy it was shown that the length of hospital and ICU admission were higher in patients with hematological malignancy compared to patients without cancer [6]. However, the length of hospital stays and ICU stay was similar between groups. In a case report of 4 patients by Bellmann-Weiler et al. All 4 patients had COVID-19 infection with comorbid hematological malignancy all recovered from COVID-19 and were discharged. The authors noted that hyperinflammatory associated organ failure may be less pronounced in hematological malignancies due to treatment related immunosuppression [7]. In an observational study by Norsa et al. of 522 patients with inflammatory bowel disease in Northern Italy it was observed that of these patients none were admitted to hospital with SARS-CoV-2 proven infection and none of the patients with IBD in this study was affected by a complicated SARS-CoV-2–related pneumonia [8]. The authors concluded that the data suggest that patients receiving immunosuppressive treatments could be at lower risk of developing severe symptomatic SARS-CoV-2 infection.

It is well-known that systemic inflammation and the resulting cytokine cascade is an important pathophysiological mechanism in the development of COVID-19 associated acute respiratory distress [9]. The resulting therapies for COVID-19 have been aimed at reducing systemic inflammation. In a study by Lee et al. On 800 patients who had a diagnosis of cancer and symptomatic COVID 19 they were unable to find that cancer patients on active cytotoxic chemotherapy were at increased risk of mortality from COVID-19 compared to those cancer patients not on chemotherapy [10].

Unfortunately, data on COVID-19 among cancer patients is still scarce, with few described cases. Along with the small sample size of only Chinese patients, there is a large amount of heterogeneity due to several cancer types and different oncologic treatments. Despite this, not all cases were fully described, with many of them already in cancer remission with no clear immunosuppression. Most of the studies included patients with a history of negative predictive factors like smoking and older age, which may explain worse outcomes of COVID-19 infection.

In conclusion, although cancer patients with COVID-19 are expected to have a more unsatisfactory outcome, data remains scarce. We are still learning from this outbreak, and international collaboration is necessary to describe better the characteristics and outcomes of COVID-19 disease among cancer patients. In this report, we show a patient with non-Hodgkin lymphoma in the middle of chemotherapy, presented a mild clinical course of COVID-19 infection. More information is still needed concerning specific cancer types and the impact of chemotherapy on COVID-19.

## Data Availability

The datasets used and/or analysed during the current study available from the corresponding author on reasonable request.

## Abbreviations

COVID-19: Corona virus disease 2019
PCR: Polymerase chain reaction
SARS-CoV-2: Severe acute respiratory syndrome-corona virus-2
BID: Twice daily
q8h: 8 Hourly
PET CT: Positron emission tomographic computed tomography scan
CD20: Cluster of differentiation 20
CHOP: Cyclophosphamide, doxorubicin, vincristine, prednisolone and methotrexate
QD: Daily
RT PCR: Reverse transcriptase polymerase chain reaction
